# Detailed Analysis of Gamma-Shielding Characteristics of Ternary Composites Using Experimental, Theoretical and Monte Carlo Simulation Methods

**DOI:** 10.3390/polym16131778

**Published:** 2024-06-24

**Authors:** Hasan Özdoğan, Yiğit Ali Üncü, Ferdi Akman, Hasan Polat, Mustafa Recep Kaçal

**Affiliations:** 1 Department of Medical Imaging Techniques, Vocational School of Health Services, Antalya Bilim University, 07190 Antalya, Turkey; 2 Department of Biomedical Equipment Technology, Vocational School of Technical Sciences, Akdeniz University, 07070 Antalya, Turkey; 3 Program of Occupational Health and Safety, Department of Property Protection and Security, Vocational School of Social Sciences, Bingöl University, 12000 Bingöl, Turkey; 4 Department of Architecture and Urban Planning, Vocational School of Technical Sciences, Bingöl University, 12000 Bingöl, Turkey; 5 Department of Physics, Arts and Sciences Faculty, Giresun University, 28100 Giresun, Turkey

**Keywords:** shielding, barite, tungsten, Monte Carlo simulation, ternary composite

## Abstract

Ionizing radiation is vital in various fields but poses health risks, necessitating effective shielding. This study investigated the photon-shielding properties of polyester-based ternary composites with barite (BaSO_4_) and tungsten (W) using experimental methods, theoretical calculations, and Monte Carlo simulations for energies between 81 keV and 1332.5 keV. WINXCOM was utilized for the theoretical predictions, and the MCNP6 and PHITS 3.22 algorithms were employed for the simulations. According to the results, the simulation, theoretical, and experimental data all closely aligned. At 81 keV, the composite containing the highest amount of tungsten (PBaW50) had the highest mass attenuation coefficient (3.7498 cm^2^/g) and linear attenuation coefficient (12.9676 cm^−1^). Furthermore, for a sample that was 1 cm thick, PBaW50 offered 99.88% protection at 81 keV and had the lowest HVL and TVL values. PBaW50 exhibited attenuation capabilities, making it appropriate for use in industrial, medical, and aerospace settings. In summary, the findings of this study underscore the potential of polyester-based composites doped with barite and tungsten as effective materials for gamma radiation shielding. The PBaW50 sample, in particular, stands out for its attenuation performance, making it a viable option for a wide range of applications where durable and efficient radiation shielding is essential.

## 1. Introduction

As technology advances, the applications of radiation also expand. Ionizing radiation poses a potential risk to human health. The interaction of radiation with the DNA molecule in the cell nucleus can damage the cell. If the damaged cell cannot be repaired, it may continue to live in a mutated form or die and be expelled from the body. These mutated DNA molecules are responsible for the harmful effects of radiation on humans.

Radiation, which has a wide range of uses from health to industry, requires adherence to certain basic principles to protect humans and the environment from its harmful effects. The fundamental principles of radiation protection are defined as time, distance, and shielding. Limiting the time and distance to the radioactive source is not always possible, especially for radiation workers. Therefore, radiation shielding is extremely important. Historically, lead has been used as a shielding material due to its high absorption capability. The walls of rooms with high radiation doses are lined with thick lead blocks, and radioactive elements are stored in heavy and thick lead containers. Radiology and nuclear medicine personnel still wear thick lead aprons to avoid radiation exposure. These aprons restrict the mobility of workers and, due to their heaviness, lead to negligence in using protective materials. Furthermore, lead is a hazardous element due to its toxic effects on human health. These drawbacks have intensified research into the production and use of alternative materials for shielding.

Intensive research and development across various disciplines have been driven by the quest for new shielding materials. A range of promising materials, including glasses [1,2,3,4], concretes [5,6,7], and composites [8,9,10], has been identified, each with unique characteristics beneficial for radiation shielding. Glasses, for instance, are valued for their dual properties of transparency and effective radiation attenuation, which are particularly advantageous in environments requiring visibility, such as medical-imaging facilities and laboratories.

Among all the materials used for radiation protection, polymers have stood out in recent years due to their affordability, ease of production, lightness, flexibility, and capability to form multi-layered structures. For this reason, many researchers have engaged in studies involving experimental and simulation techniques on the radiation permeability of various polymers. Nagaraja et al. have investigated the gamma-shielding performances of polymers such as polystyrene, polypropylene, polytetrafluoroethylene (PTFE), polyvinyl chloride (PVC), and polychlorotrifluoroethylene (PCTFE) up to an energy of 1.330 MeV and found that PCTFE’s radiation shielding property is superior to the others [11]. In another study, the photon absorption properties of epoxy resin with carbopol polymers, used in breast phantoms of different densities, were examined at low energies, and it was found that the density differences did not significantly affect the radiation permeability [12]. The radiation permeabilities of synthetic polymers, including polyethylene (PE), polystyrene (PS), polycarbonate (PC), polyvinyl alcohol (PVA), polyvinyl chloride (PVC), polyethylene terephthalate (PET), polyvinylpyrrolidone (PVP), polytetrafluoroethylene (PTFE), polypropylene (PP), and polymethyl methacrylate (PMMA), were investigated by Mirji et al. between the energies of 14.4 and 1332 keV [13]. In another study, barite-based ternary polymer composites containing bismuth were explored, showing significant gamma ray-shielding characteristics. Notably, a composite designated BaBi50 was highlighted for its superior shielding effectiveness. Additionally, the incorporation of gadolinium (III) sulfate was shown to enhance the gamma radiation-shielding capabilities, while adding polyacrylonitrile improved the neutron-shielding properties [14]. Research on zircon- and corundum-based composites primarily focused on their gamma ray-shielding properties. The findings indicated that composites with heavy metals offered better gamma ray attenuation, whereas those with lighter metals were more effective against fast neutrons [15]. Further investigations into SiC/Gd2O3/6061Al composites demonstrated that the SiC content significantly influenced both the shielding properties and mechanical strength of these materials. These composites were notably proficient in neutron shielding, with the gamma-shielding efficacy varying based on the composition [16]. A study reported remarkable enhancements in both the neutron- and gamma-shielding properties (200–280% and 14–52% increases, respectively) by adding h-BN and Gd2O3 nanoparticles to HDPE ternary composites. It was noted that an increase in the filler content led to higher absorption rates for neutron and gamma fluxes [17]. Lastly, research into ternary nanocomposites containing PbO revealed a 47% improvement in gamma-shielding effectiveness compared to concrete, with a positive correlation established between the PbO content and the mass attenuation coefficient. Furthermore, core-shell-structured particles were introduced into ternary composites, yielding not only improved tensile strength but also promising neutron- and gamma-shielding properties [18,19].

In our previous study, the radiation absorption properties for gamma, neutron, and charged particle radiation of polyester-based composites filled with barite and/or tungsten boride were calculated using experimental GEANT4, FLUKA, and MCNP Monte Carlo simulation codes. It was found that the polymer containing the highest amount of tungsten boride had the highest radiation absorption capacity [20]. 

In this study, the radiation permeability of ternary composites was investigated for radiation shielding due to their lower cost, ease of production, and lighter weight compared to lead. The photon-shielding performances of polyester barite tungsten composites were investigated using experimental, theoretical, and Monte Carlo simulations. The theoretical calculations were conducted using the WINXCOM [21] data center, while the Monte Carlo simulations were performed using the PHITS 3.22 [22] and MCNP6 [23] codes.

## 2. Materials and Methods

**Figure 1:**
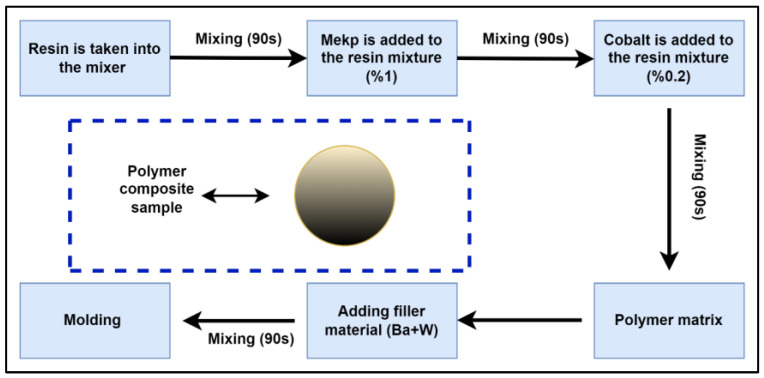
Polymer composite production stages.

In the production of polymer composite samples, the polymer matrix was initially prepared. In this process, MEKP (methyl ethyl ketone peroxide) was used as the hardener and cobalt-based organic peroxide as the accelerator to complete the reaction of the unsaturated polyester polymer resin, branded as the Turkuaz TP100 type (Turkuaz Polyester, Kocaeli, Turkey). The resin was placed in a 19 L capacity laboratory-scale mixer and mixed for 90 s. After this initial mixing, 1% MEKP was added in the predetermined proportion of the polymer quantity, followed by an additional 90 s of mixing. Then, 0.2% cobalt was added and the mixture was again mixed for 90 s to obtain the polymer matrix. A control sample was produced by adding filler material (barite) to the resulting polymer matrix in the ratio of 50% polyester resin to 50% barite, and it was mixed for 90 s for homogeneity. Five groups of polymer composite samples, excluding the control sample, were obtained by substituting barite at specific proportions (10, 20, 30, 40, and 50%) with tungsten. The production stages of the polymer composites are shown in Figure 1.

**Figure 2:**
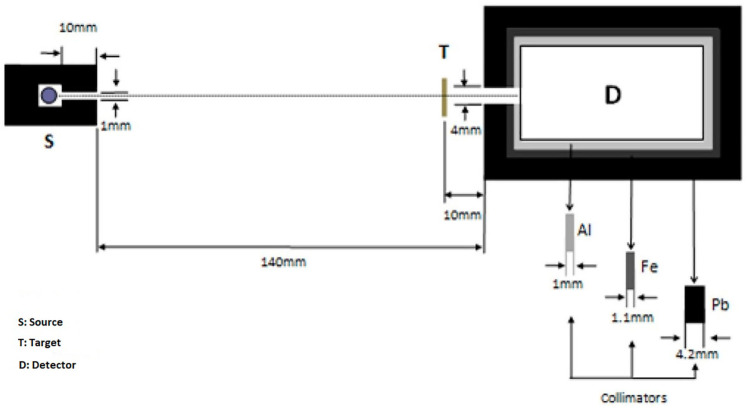
Schematic representation of the experimental geometry.

The experimental geometry used to determine the gamma ray-shielding characteristics of the produced composites containing polyester, barite, and tungsten is presented in Figure 2. As shown in Figure 2, the experimental system consists of radioactive sources, produced composite material, a detector, and various collimators. Ba-133, Na-22, Cs-137, and Co-60 point sources were chosen as the radioisotope sources, with the energies released from these sources ranging from 81.0 to 1332.5 keV gamma energies. Comprehensive information about these sources was provided in the previous study [24]. A lead collimator was used to obtain the parallel photons emitted from these sources. A high-purity germanium (HPGe) detector was used to perform the experimental measurements. This detector, an Ortec brand GEM-SP7025P4-B model (Ortec/Ametek, Oak Ridge, TN, USA), has an active crystal radius of 35 mm. Additionally, this detector has a guaranteed resolution of 1800 eV at 1333 MeV, 585 eV at 122 keV, and 380 eV at 5.9 keV. The detector was accompanied by a DSPEC-JR 2.0V.046 model multi-channel analyzer (Ortec/Ametek, Oak Ridge, TN, USA), and a 2600 V positive-polarity high-voltage source was used. During the measurements, the detector was maintained at −196 °C in liquid nitrogen to ensure minimal electronic noise. The collimators used between the source–target and target–detector helped establish a narrow beam geometry, while the sandwich collimator (Pb-Fe-Al) around the detector prevented detection of environmental radiation. The measurement times were adjusted so that the area under each energy peak was generally at least 10,000 counts. The areas of these peaks were determined with the MAESTRO interface. The radiation intensities in the absence of the target (*I*_0_) and in the presence of the target (*I*), obtained as a result of the measurements, were used to determine the gamma ray-shielding characteristics of the produced composites.

While determining the experimental uncertainties, the uncertainties in the mass attenuation coefficient were first determined using Equation (1) [25]. Since other parameters depend on the mass attenuation coefficient, the uncertainties in these parameters were obtained using the root mean square distribution principle of uncertainty.

(1) ∆μρ=1ρx∆I0I02+∆II2+ln∆II2∆ρxρx2

In Equation (1), *I*, *I*_0_ and *ρ_x_* indicate the radiation intensity in the presence of the sample, the radiation intensity in the absence of the sample and the mass per unit area, respectively, while ΔI, Δ*I*_0_ and *ρ_x_* indicate the uncertainties in them. In the performed measurements, they were determined as approximately Δ*I* < 3%, Δ*I*_0_ < 2% and Δ*ρx* < 2%.

After the gamma ray experiments were conducted, the experimental results were compared with the theoretical calculations and Monte Carlo simulations. The theoretical calculations were performed using the absorption coefficients obtained from the WINXCOM data center and the equations given below. The linear attenuation coefficient (LAC), denoted as *μ*, represents the reduction resulting from the interactions between the highly energetic photons emitted from any radioactive source and an absorber. It can be computed by modifying the Beer–Lambert equation as follows (Equation (2)):

(2) $$\mu =-\frac{\ln\frac{I}{I_{0}}}{x}$$

where *x*, *I*_0_, and *I* denote the thickness of the absorber and the photon intensities before and after attenuation, respectively. It is imperative to correlate the LAC value with the density of the associated material. In this context, the recognized mixture rule validates the photon attenuation of the absorber for the precision of the fabricated materials, as given by Equation (3):

(3) $$\mu_{m}=\frac{\mu }{\rho }=\sum w_{i}{\left(\frac{\mu }{\rho }\right)}_{i}$$

where *w* is the mass ratio of the elements within the polymer. The mass attenuation coefficient (MAC) is defined as the ratio of the linear attenuation coefficient to the density of the material. The MAC is a fundamental physical quantity that characterizes a material’s ability to absorb radiation at a specific energy. The coefficient is typically measured in units of cm^2^/g and represents the likelihood of a material absorbing radiation as it traverses through.

A higher MAC indicates that the material is more effective in absorbing radiation at a given energy. This parameter plays a crucial role in various fields, such as medical imaging, radiation therapy, nuclear physics, and industrial. Understanding the mass attenuation coefficient is essential for characterizing materials and comprehending the behavior of radiation within them. Researchers and practitioners use this coefficient to optimize imaging techniques, dose calculations, and radiation-shielding strategies.

The half-value layer (HVL), another valuable shielding parameter, is defined as the thickness of a substance required to reduce the radiation levels to half the initial photon counts. Its calculation involves the linear attenuation coefficients, as shown in Equation (4):

(4) $$HVL=\frac{\ln\;2}{\mu }$$

The mean free path (MFP) represents the average distance that radiation traverses before a photon interacts with shielding materials, which is computed as follows:

(5) MFP=1/μ

The ratio *σ_a_/σ_e_* is used to evaluate the value of *Z_eff_*, where *σ_a_* and *σ_e_* are the atomic and electronic cross-sections, respectively, calculated using the μm values. The energy absorption (EABF) and exposure build-up factors (EBF) are two other important parameters that help in examining the radiation-shielding properties in wide-beam geometry.

The term known as the tenth value layer (TVL) refers to the thickness of a material that reduces the intensity of a particular type of radiation to one-tenth. The TVL is used to evaluate the absorption properties of radiation and understand a material’s ability to absorb radiation. In this context, the TVL values are usually given for X-rays or gamma rays at a specific energy and provide information about the depth of the radiation within a material as follows:

(6) $$TVL=\frac{\ln\;10}{\mu }$$

The effective electron density (*Neff*) is calculated as follows:

(7) $$Neff=\frac{N_{A}n_{t}Z_{eff}}{\sum_{i}n_{i}A_{i}}$$

where $\sum_{i}n_{i}A_{i}$ is the molecular weight, *n_t_* is the total number of atoms, *N_A_* is Avogadro’s number, and *Z_eff_* is the effective atomic number.

The radiation protection efficiency (RPE), also known as the dose reduction factor (DRF), of a material is determined as follows;

(8) $$RPE=\left(1-\frac{I}{I_{0}}\right)\times 100$$

where 100 represents the *RPE* (%) of the coefficient of percent efficiency (%). In Equation (8), *I*_0_ and I denote the photon intensities before and after attenuation, respectively.

**Figure 3:**
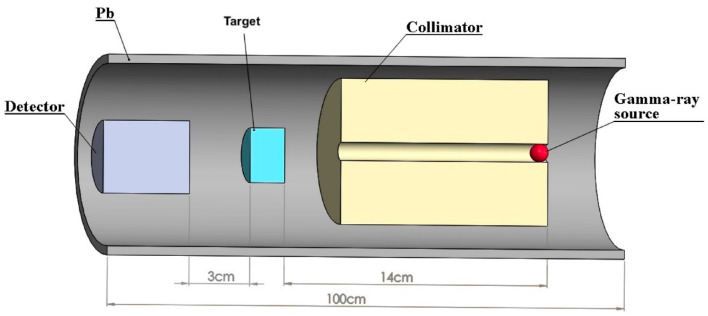
Schematic representation of the simulation geometry.

According to the reports of the National Council on Radiation Protection and Measurements, the use of Monte Carlo simulations is necessary when deviating from standard materials in radiation shielding [26,27,28]. In our study, alongside the theoretical and experimental calculations, we also conducted computations using Monte Carlo simulations. These simulations, along with theoretical calculations, serve to test and replicate the accuracy of experimental results when necessary. Experimental data aligning with the outcomes of theoretical efforts and simulations are deemed more reliable. We utilized a simplified narrow-beam setup consisting of a point gamma ray source and a sample slab, as outlined in Figure 3. It is important to note that the shielding material was considered homogeneous throughout these simulations. The point gamma ray source and the detector were encased in lead blocks to ensure effective shielding. The point sources used in the experiment, Ba-133, Na-22, Cs-137, and Co-60, were defined in the simulation in the same manner as the point sources emitting gamma rays at the same energies. The source-to-sample distance was set at 14 cm, while the detector was positioned 3 cm away from the sample. Simulations were carried out individually for scenarios with and without the shielding material, just as in the experimental procedure. The photon influx, measured in MeV cm^2^s^−1^ by the detector, was recorded using different methods: the F4 tally in MCNP6 and the T-Track tally in PHITS 3.22. To minimize the error rate, the Monte Carlo simulations were performed 10 million times. As a result, the tally uncertainty was found to be 0.001 by the simulation codes. These measurements were essential for applying the Beer–Lambert law to determine the linear attenuation coefficient, factoring in the energy specificity and material thickness.

## 3. Results

**Figure 4:**
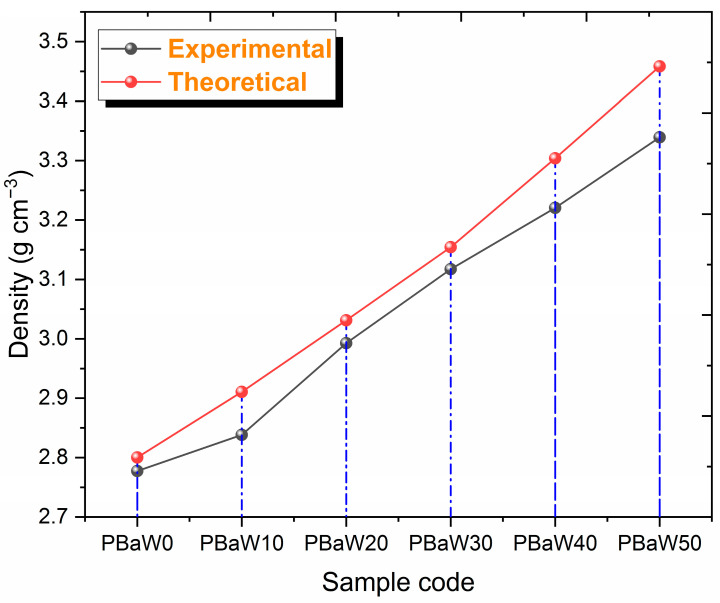
The change in the density with an increasing amount of tungsten.

The gamma radiation-shielding characteristics of the produced composites by weight replacement of barite (BaSO4) and tungsten (W) materials, keeping the amount of polyester constant, were investigated in the gamma ray energy range from 81.0 to 1332.5 keV. While experimental measurements were carried out with the help of the mechanism presented in Figure 2, simulation studies were also carried out with the help of the geometry in Figure 3 and the chemical contents presented in Table 1. The gamma radiation-shielding capacities of the produced ternary composites were investigated with the help of the µ, µ/ρ, HVL, MFP, TVL, Z_eff_, NE and RPE parameters. The sample densities used in the calculation of some gamma radiation-shielding parameters are presented in Table 1 and Figure 4. The densities of the produced ternary composites were determined experimentally with the help of Archimedes’ principle [29] and theoretically with the help of the principle of determining the densities of mixtures [30]. As seen in Figure 4, it was observed that the densities of the composites increase with an increasing tungsten amount or decreasing barite amount. The observed difference between the theoretical and experimental densities can be attributed to several factors: purity, precision of the instruments, temperature and pressure conditions, and structural properties of the material. Despite these potential sources of discrepancy, it is important to note that the maximum observed difference is a modest 3.44%, indicating a generally good agreement between the theoretical and experimental results. Therefore, it can be said that the experimental and theoretical intensities are compatible with each other. This harmony shows that the formed voids in the produced composites are small. The experimental density values were used for the experimental calculations, and the theoretical density values were used for the theoretical and simulation calculations.

**Figure 5:**
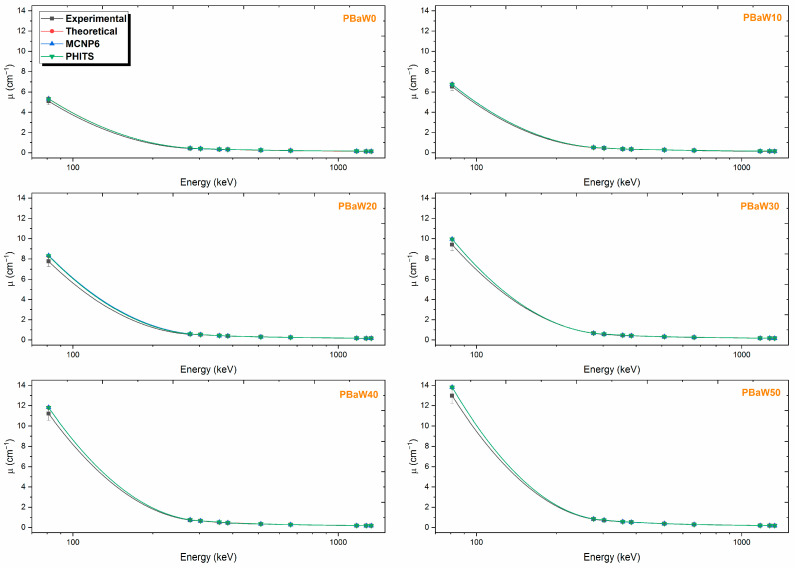
The comparative representation of the experimental, theoretical and simulation µ results.

The linear attenuation coefficient, the results of which are listed in Table 2 and the variation of which with gamma rays is visually presented in Figure 5, is an important parameter that provides reliability in determining the gamma radiation-shielding capacity and the usability of the shielding material. With this parameter, other parameters such as the HVL, MFP and TVL, which indicate the material thickness that reduces the radiation intensity to certain levels, can be derived. The data are not clearly visible in Figure 5, which indicates how well the experimental data, simulations, and theoretical calculations align. The reliability of the calculations can be clearly seen from the graph. As shown in Figure 5, there is harmony between the linear attenuation coefficients determined by the experimental, theoretical, MCNP6, and PHITS approaches. This suggests that the MCNP6 and PHITS simulation codes provide reliable results for the investigated composites. Again, from Figure 5, it is seen that the linear attenuation coefficient decreases exponentially with increasing gamma ray energy and increases with an increasing tungsten amount or decreasing barite amount. The exponential change in this parameter is due to the different dominant interactions of varying gamma energies with matter. There are different dominant interaction processes between the material and the gamma rays in the low (E < 125 keV), medium (125 < E < 1.022 keV) and high (E > 1.022 keV) energy regions. Photoelectric effect, Compton scattering, and pair production processes are dominant in these regions, respectively. Since the photoelectric absorption cross-section is proportional to E^−3.5^, the sudden decrease in the low-energy region can be interpreted with this cross-section. Since the Compton scattering cross-section and the pair production cross-section are proportional to E^−1^ and E, respectively, slight changes in the medium- and high-energy regions can be interpreted with these cross-sections. Since it was seen that the gamma ray-shielding capacity improved with an increasing tungsten amount, it was seen from the linear attenuation coefficient results that the coded PBaW50 composite containing 50% tungsten had a superior gamma ray-shielding capacity compared to the others.

**Figure 6:**
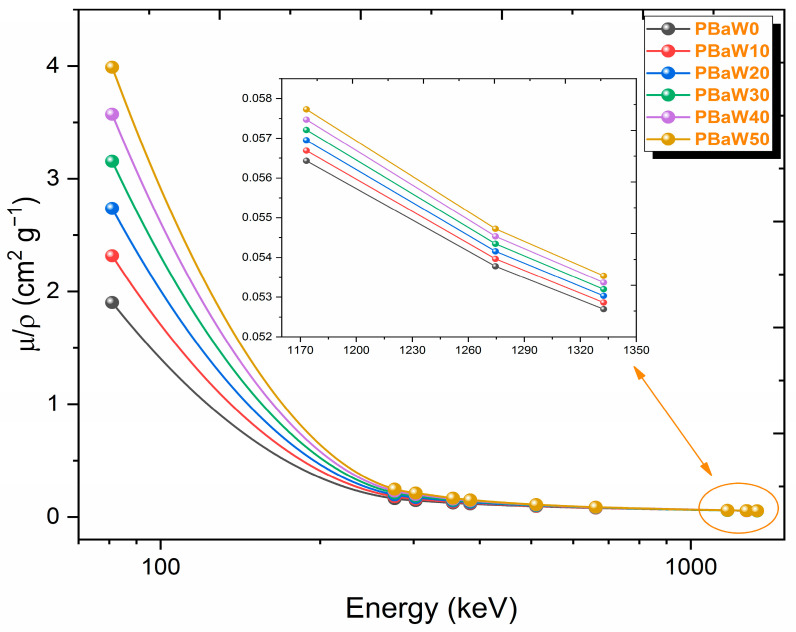
The variation of the µ/ρ results (obtained by experiment) with the photon energy.

The mass attenuation coefficient, which can be easily found by dividing the linear attenuation coefficient by the density of the material and is independent of the material density, is the second investigated gamma ray-shielding characteristic parameter. Although this parameter provides information about gamma ray shielding, it may cause errors in the selection of the appropriate material in terms of the usability, as it is independent of the density. The change graph of the mass attenuation coefficients of the composites containing polyester resin, barite and tungsten with the gamma ray energy is presented in Figure 6. According to Figure 6, like the linear attenuation coefficient results, it was observed that the mass attenuation coefficient decreases with increasing energy and increases with an increasing tungsten amount. This exponential decrease can be attributed to the energy dependence of the cross-sections for photoelectric absorption, Compton scattering, and pair production, similar to the behavior seen with the linear attenuation coefficient. Indeed, a decrease in the mass attenuation coefficient with increasing energy is expected. Within the energy spectrum under consideration, the photoelectric effect dominates at energies below 125 keV, Compton scattering occurs between 125 keV and 1.022 MeV, and pair production becomes significant above 1.022 MeV. The total attenuation coefficient is determined by the cumulative contributions of each of these interactions, all of which are inversely proportional to the energy. Consequently, as the energy increases, the likelihood of these interactions decreases, leading to a reduction in the mass attenuation coefficients. Therefore, to effectively stop radiation at higher energies, materials must be designed to be thicker. Considering the mass attenuation coefficient results, it was observed that the sample that did not contain tungsten, coded as PBaW0, had the worst gamma ray-shielding capacity, while the composite that contains 50% tungsten, coded as PBaW50, had the best gamma ray-shielding capacity. According to the mass attenuation coefficient results, PBaW50 is 2.1 times better than PBaW0 at 81.0 keV and 1.1 times better than PBaW0 at 661.7 keV in terms of gamma ray shielding.

**Figure 7:**
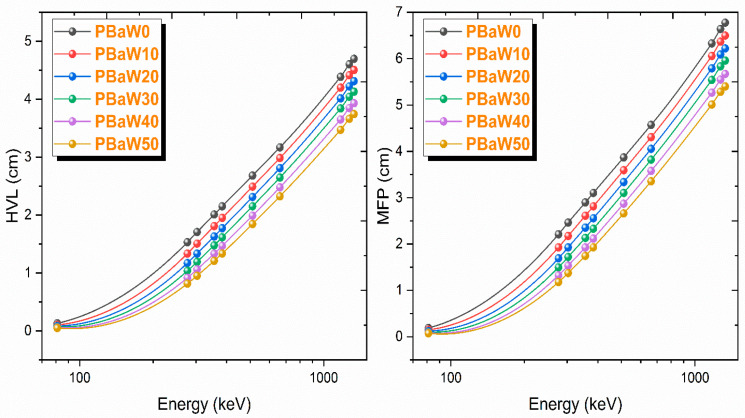
The variation of the HVL and MFP results (obtained by experiment) with the photon energy.

**Figure 8:**
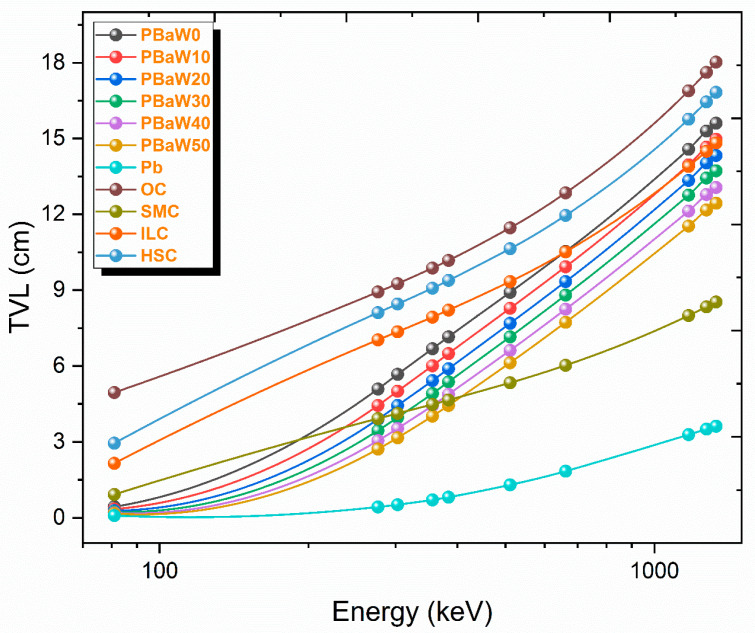
The comparative TVL results (obtained by experiment) versus the photon energy of the produced ternary composites and some materials commonly used for gamma ray shielding.

The HVL, MFP and TVL parameters, which are related to the thickness of the gamma ray-shielding material and can be determined with the help of the linear reduction coefficient, with their lower values indicating that gamma ray shielding is better in this respect, have been determined for the produced composites. These parameters also provide reliability in material selection for gamma ray shielding. HVL, MFP and TVL are known as material thicknesses that reduce the gamma ray intensity by 50%, 63.2% and 90%, respectively. While the variation of the HVL and MFP results with the gamma ray energy is presented in Figure 7, the TVL results of the produced composites, lead and some concrete types [31] are presented in Table 3 and Figure 8. As can be clearly seen from Figure 7 and Figure 8, it has been observed that the HVL, MFP and TVL values increase with increasing energy and decrease with an increasing tungsten amount. According to these parameters, the HVL, MFP and TVL results are listed as PBaW0 > PBaW10 > PBaW20 > PBaW30 > PBaW40 > PBaW50. As can be seen from this ranking, it is obvious that the sample coded as PBaW50 is a better gamma ray-shielding material than the others. When the PBaW0 and PBaW50 composites are examined considering the HVL parameter, PBaW50 has 159.2%, 36.3% and 25.5% higher gamma ray-shielding capacity than PBaW0 at 81.0, 661.7 and 1332.5 keV gamma ray energies, respectively. In Table 3 and Figure 8, the TVL values of the produced composites and the TVL results of lead and some concrete types, such as ordinary, steel–magnetite, ilmenite–limonite, and hematite–serpentine, are presented comparatively. According to Figure 8, it was observed that all the produced composites show superior gamma ray-shielding properties to ordinary and hematite–serpentine concretes, and these composites have worse gamma ray-shielding properties than lead. If the TVL results of lead and ordinary, steel–magnetite, ilmenite–limonite and hematite–serpentine concretes are compared with the sample coded as PBaW50 composite, which has the best gamma ray-shielding feature among the produced composites, PBaW50 is 0.52–0.16 times higher than lead, 29.65–1.45 times higher than ordinary concrete, 5.52–0.68 times higher than steel–magnetite concrete, 12.87–1.19 times higher than ilmenite–limonite concrete and 17.63–1.35 times higher than hematite–serpentine concrete in terms of the gamma ray-shielding capacity.

**Figure 9:**
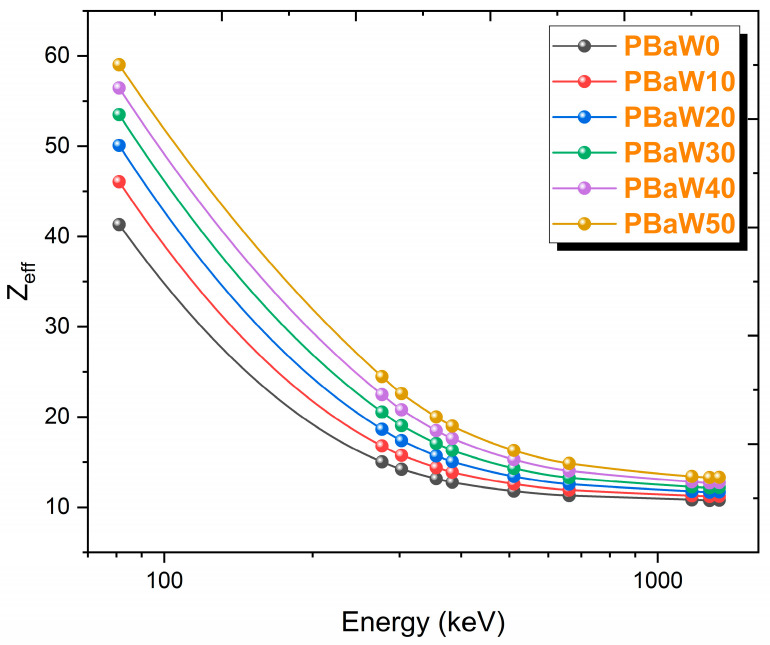
The variation of the Z_eff_ results (obtained by theory) with the photon energy.

**Figure 10:**
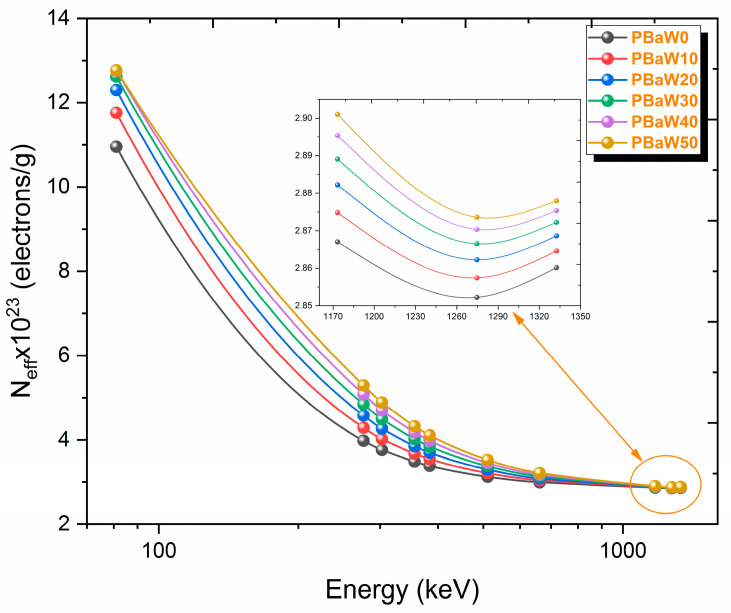
The variation of the N_eff_ (obtained by theory) results with the photon energy.

The results of the effective atomic number parameter used in materials other than elements and the effective electron density parameter, which expresses the number of electrons in the unit mass, are shown in Figure 9 and Figure 10. Since there is a direct proportion between these two parameters, their changes with the gamma rays show similar trends. Like the mass and linear attenuation coefficients, it has been observed that these parameters decrease with increasing gamma ray energy and increase with an increasing tungsten amount. At 81.0 keV gamma ray energy, the effective atomic numbers of the PBaW0, PBaW10, PBaW20, PBaW30, PBaW40 and PBaW50 composites were determined as 41.32, 46.05, 50.07, 53.49, 56.44 and 59.02, while the effective electron densities were determined as 10.95, 11.76, 12.30, 12.61, 12.76 and 12.77 (×10^23^) electrons/g, respectively. As can be seen from these data, the PBaW50 composite is a better gamma ray-shielding material than the others.

**Figure 11:**
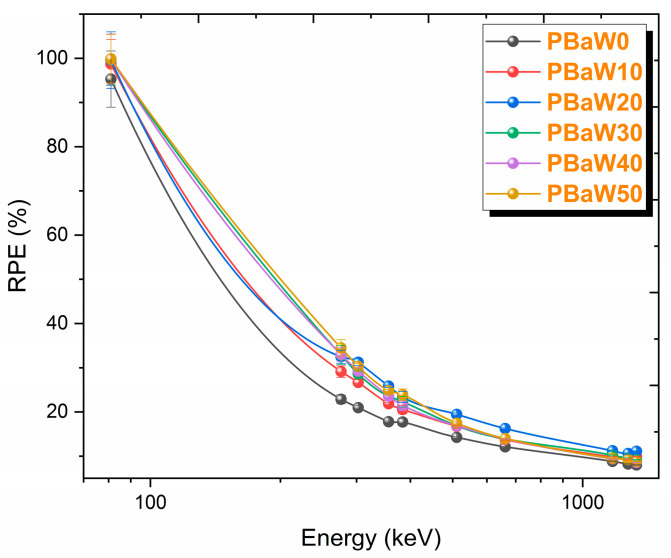
The variation of the RPE results (obtained by experiment) with the photon energy.

The last parameter obtained to determine the gamma ray-shielding capacity of the produced composites is the RPE. This parameter was obtained with the help of the gamma ray intensities measured in the absence (I_0_) and presence (I) of the sample at 1 cm sample thickness. It can be seen from Figure 11 that this parameter decreases with increasing gamma ray energy and increases with an increasing tungsten amount. Since the produced composites have very high RPE values at low energies, it has been observed that the produced composites at low energies are good gamma ray-shielding materials. Like other parameters, according to the RPE results, the coded PBaW50 composite was found to be a better gamma ray-shielding material than the others. The RPE values for the materials studied, at a thickness of 1 cm and energy of 81 keV, are found to be 95.29% for PBaW0, 98.72% for PBaW10, 99.60% for PBaW20, 99.67% for PBaW30, 99.77% for PBaW40, and 99.88% for PBaW50. When calculated using Equation (7) for the same energy and thickness, the RPE value for pure lead is approximately 100%. These results suggest that the proposed materials, especially PBaW40 and PBaW50, can provide a dose reduction similar to that of pure lead at the same energy and thickness.

## 4. Conclusions

Ionizing radiation holds a significant place in scientific research due to its complex characteristics and health risks. It can interact with biological systems, leading to harmful effects such as DNA damage and cancer. Understanding and mitigating these risks is crucial across many fields. Despite its dangers, radiation is indispensable in areas like medicine, where it is used in diagnostic-imaging techniques such as X-rays, CT scans, and nuclear medicine, aiding in diagnosis, treatment guidance, and saving lives. In industry, radiation is essential for sterilization, material testing, and quality control, enhancing safety and efficiency. Academically, radiation research drives advancements in nuclear physics, chemistry, and environmental science, contributing to fundamental knowledge and practical application. Additionally, nuclear power generation offers a significant, low-carbon energy source. The challenge is to harness these benefits while minimizing the risks, which involves effective radiation shielding to protect individuals and the environment. In radiation protection, the ALARA principle, standing for “As Low As Reasonably Achievable”, is vital for minimizing exposure. ALARA emphasizes reducing radiation exposure to the lowest possible levels considering the circumstances. It is applied through three key principles: time, distance, and shielding. Mastering and implementing these principles are essential to protect people and the environment from the harmful effects of ionizing radiation.

Composites using polyester-based fillers are well established as a cost-effective, durable, and efficient photon absorption solution. These materials have the potential to considerably improve the shielding properties of a variety of applications. In this study, ternary composites with various component ratios were initially produced. Subsequently, the photon-shielding properties of ternary composites comprising polyester, barite, and tungsten were evaluated using experimental methods, theoretical calculations, and Monte Carlo simulations. The range of energies analyzed extended from 81 keV to 1332.5 keV. Theoretical predictions were performed using the WINXCOM computer program, while the MCNP6 and PHITS 3.22 codes facilitated the Monte Carlo simulations. The obtained results can be summarized as follows.

(1) The experimental findings closely align with both the theoretical and Monte Carlo simulation results. The Monte Carlo simulation and theoretical calculations can be used in the absence of experimental facilities.
(2) The linear attenuation coefficients at 81 keV photon energy were experimentally found to be 5.0840 ± 0.7039 cm^−1^ for PBaW0, 6.5264 ± 0.7987 cm^−1^ for PBaW10, 7.7616 ± 0.5224 cm^−1^ for PBaW20, 9.4154 ± 0.5825 cm^−1^ for PBaW30, 11.2051 ± 0.6836 cm^−1^ for PBaW40, and 12.9676 ± 0.7597 cm^−1^ for PBaW50. It was determined that PBaW50, which has the highest W ratio, has the highest linear attenuation coefficient value.
(3) The mass attenuation coefficients at 81 keV photon energy were experimentally found to be 1.8156 cm^2^/g for PBaW0, 2.2422 cm^2^/g for PBaW10, 2.5605 cm^2^/g for PBaW20, 2.9848 cm^2^/g for PBaW30, 3.3914 cm^2^/g for PBaW40, and 3.7498 cm^2^/g for PBaW50.
(4) The HVL results at 81 keV photon energy were experimentally found to be 0.1363 cm for PBaW0, 0.1062 cm for PBaW10, 0.0893 cm for PBaW20, 0.0736 cm for PBaW30, 0.0619 cm for PBaW40, and 0.0535 cm for PBaW50. The TVL results at 81 keV photon energy were experimentally found to be 0.4529 cm for PBaW0, 0.3528 cm for PBaW10, 0.2967 cm for PBaW20, 0.2446 cm for PBaW30, 0.2055 cm for PBaW40, and 0.1776 cm for PBaW50. The polymer with the lowest HVL and TVL values was identified as PBaW50.
(5) The RPE values for 1 cm thickness of the materials were found to be as follows: 95.29% for PBaW0, 98.72% for PBaW10, 99.60% for PBaW20, 99.67% for PBaW30, 99.77% for PBaW40, and 99.88% for PBaW50.
(6) Considering the LAC, MAC, HVL, TVL, and RPE values, it was concluded that PBaW50, the polymer with the highest W ratio, is the most effective material for radiation shielding among those studied. PBaW50 exhibits the highest LAC, MAC, and RPE values, along with the lowest HVL and TVL values, making it the best choice for radiation shielding. Consequently, the PBaW50 sample demonstrates outstanding attenuation performance and could be considered for applications requiring durable and lightweight materials in sectors such as industry, medicine, and aerospace. By incorporating high-atomic-number elements like tungsten (W) into new materials, more effective radiation shielding can be achieved. Before producing shielding materials, they should be designed according to their intended use and purpose, considering factors such as the weight, importance, and cost.

## Figures and Tables

**Table 1:** The chemical compositions and densities of the ternary composites produced with polyester resin, barite and tungsten.

Sample Code	Chemical Composition (%)							Density (g/cm^3^)	
	H	C	O	S	Co	Ba	W	Exp.	Theo.
PBaW0	0.9025	12.1200	28.9989	10.9746	0.0024	47.0017	0.0000	2.7775	2.8002
PBaW10	0.9025	12.1200	26.8182	9.8820	0.0024	42.3224	7.9525	2.8383	2.9107
PBaW20	0.9025	12.1200	24.6181	8.7797	0.0024	37.6013	15.9760	2.9927	3.0313
PBaW30	0.9025	12.1200	22.4277	7.6822	0.0024	32.9012	23.9640	3.1174	3.1544
PBaW40	0.9025	12.1200	20.2373	6.5848	0.0024	28.2010	31.9521	3.2206	3.3040
PBaW50	0.9025	12.1200	18.0469	5.4873	0.0024	23.5008	39.9401	3.3392	3.4583

**Table 2:** The linear attenuation coefficient results (cm^−1^) obtained with the experimental, theoretical, MCNP6 and PHITS for the investigated ternary composites.

**Energy (keV)**	**PBaW0**				**PBaW10**			
	**Experimental**	**Theo.**	**MCNP6**	**PHITS**	**Experimental**	**Theo.**	**MCNP6**	**PHITS**
81.0	5.0840 ± 0.7039	5.3229	5.3151	5.3173	6.5264 ± 0.7987	6.7428	6.7406	6.7340
276.4	0.4315 ± 0.3537	0.4528	0.4552	0.4545	0.5156 ± 0.3873	0.5192	0.5202	0.5199
302.9	0.3912 ± 0.0207	0.4063	0.4074	0.4097	0.4645 ± 0.0254	0.4602	0.4629	0.4629
356.0	0.3257 ± 0.0115	0.3449	0.3460	0.3475	0.3692 ± 0.0143	0.3832	0.3842	0.3862
383.9	0.3241 ± 0.0073	0.3221	0.3228	0.3247	0.3446 ± 0.0085	0.3551	0.3562	0.3581
511.0	0.2555 ± 0.0157	0.2585	0.2597	0.2610	0.2743 ± 0.0175	0.2782	0.2793	0.2792
661.7	0.2144 ± 0.0065	0.2188	0.2198	0.2201	0.2213 ± 0.0071	0.2321	0.2329	0.2337
1173.2	0.1533 ± 0.0047	0.1580	0.1591	0.1588	0.1543 ± 0.0048	0.1650	0.1663	0.1663
1274.5	0.1425 ± 0.0036	0.1506	0.1520	0.1520	0.1472 ± 0.0036	0.1571	0.1585	0.1588
1332.5	0.1387 ± 0.0037	0.1476	0.1490	0.1489	0.1563 ± 0.0037	0.1539	0.1550	0.1550
**Energy (keV)**	**PBaW20**				**PBaW30**			
	**Experimental**	**Theo.**	**MCNP6**	**PHITS**	**Experimental**	**Theo.**	**MCNP6**	**PHITS**
81.0	7.7616 ± 0.5224	8.2937	8.3414	8.2886	9.4154 ± 0.5825	9.9479	9.9524	9.9492
276.4	0.5528 ± 0.0278	0.5918	0.5956	0.5920	0.6571 ± 0.0453	0.6688	0.6691	0.6684
302.9	0.5275 ± 0.0169	0.5192	0.5246	0.5215	0.5559 ± 0.0175	0.5815	0.5830	0.5831
356.0	0.4208 ± 0.0096	0.4251	0.4291	0.4275	0.4435 ± 0.0101	0.4693	0.4713	0.4713
383.9	0.3752 ± 0.0214	0.3911	0.3937	0.3939	0.4166 ± 0.0241	0.4290	0.4307	0.4317
511.0	0.3044 ± 0.0080	0.2997	0.3026	0.3023	0.3047 ± 0.0077	0.3222	0.3222	0.3240
661.7	0.2494 ± 0.0055	0.2466	0.2487	0.2481	0.2469 ± 0.0054	0.2618	0.2627	0.2641
1173.2	0.1676 ± 0.0039	0.1726	0.1745	0.1742	0.1775 ± 0.0041	0.1805	0.1811	0.1822
1274.5	0.1574 ± 0.0040	0.1642	0.1667	0.1666	0.1641 ± 0.0042	0.1714	0.1728	0.1739
1332.5	0.1657 ± 0.0037	0.1608	0.1625	0.1620	0.1593 ± 0.0035	0.1678	0.1687	0.1694
**Energy (keV)**	**PBaW40**				**PBaW50**			
	**Experimental**	**Theo.**	**MCNP6**	**PHITS**	**Experimental**	**Theo.**	**MCNP6**	**PHITS**
81.0	11.2051 ± 0.6836	11.7990	11.8124	11.8009	12.9676 ± 0.7597	13.7941	13.8048	13.7960
276.4	0.7363 ± 0.0371	0.7559	0.7553	0.7553	0.8134 ± 0.0466	0.8492	0.8465	0.8484
302.9	0.6357 ± 0.0201	0.6524	0.6542	0.6539	0.6937 ± 0.0223	0.7281	0.7302	0.7293
356.0	0.4940 ± 0.0112	0.5198	0.5208	0.5222	0.5490 ± 0.0124	0.5735	0.5746	0.5754
383.9	0.4443 ± 0.0255	0.4724	0.4743	0.4743	0.5257 ± 0.0305	0.5186	0.5196	0.5207
511.0	0.3394 ± 0.0086	0.3482	0.3490	0.3505	0.3692 ± 0.0097	0.3758	0.3764	0.3778
661.7	0.2750 ± 0.0060	0.2795	0.2805	0.2815	0.2897 ± 0.0063	0.2982	0.2984	0.3012
1173.2	0.1815 ± 0.0042	0.1899	0.1907	0.1913	0.1927 ± 0.0044	0.1996	0.2002	0.2012
1274.5	0.1776 ± 0.0045	0.1802	0.1817	0.1822	0.1854 ± 0.0047	0.1892	0.1907	0.1913
1332.5	0.1649 ± 0.0037	0.1763	0.1770	0.1777	0.1773 ± 0.0039	0.1851	0.1859	0.1868

**Table 3:** The comparative TVL results (cm) of the produced ternary composites and some materials commonly used for gamma ray shielding with the aid of WINXCOM.

Energy (keV)	PBaW0	PBaW10	PBaW20	PBaW30	PBaW40	PBaW50	Pb	Ordinary concrete (OC)	Steel–Magnetite Concrete (SMC)	Ilmenite–Limonite Concrete (ILC)	Hematite–Serpentine Concrete (HSC)
81.0	0.4326	0.3415	0.2776	0.2315	0.1952	0.1669	0.0865	4.9501	0.9213	2.1476	2.9432
276.4	5.0855	4.4346	3.8908	3.4431	3.0463	2.7116	0.4234	8.9257	3.9187	7.0256	8.1027
302.9	5.6674	5.0030	4.4352	3.9594	3.5297	3.1625	0.5132	9.2519	4.1217	7.3460	8.4462
356.0	6.6763	6.0083	5.4165	4.9064	4.4302	4.0146	0.7061	9.8669	4.4810	7.9239	9.0736
383.9	7.1477	6.4844	5.8877	5.3672	4.8740	4.4396	0.8117	10.1732	4.6515	8.2025	9.3792
511.0	8.9078	8.2777	7.6840	7.1475	6.6122	6.1270	1.2990	11.4622	5.3326	9.3363	10.6362
661.7	10.5252	9.9212	9.3360	8.7966	8.2378	7.7225	1.8411	12.8426	6.0255	10.5125	11.9547
1173.2	14.5711	13.9544	13.3378	12.7590	12.1267	11.5336	3.2851	16.8846	7.9890	13.8888	15.7661
1274.5	15.2909	14.6594	14.0268	13.4324	12.7802	12.1679	3.5038	17.6164	8.3347	14.4900	16.4491
1332.5	15.6042	14.9646	14.3234	13.7209	13.0588	12.4370	3.6144	18.0227	8.5249	14.8221	16.8272

## Data Availability

The original contributions presented in the study are included in the article, further inquiries can be directed to the corresponding author.
